# Development and Verification of Prognostic Prediction Models for Patients with Brain Trauma Based on Coagulation Function Indexes

**DOI:** 10.1155/2022/3876805

**Published:** 2022-07-26

**Authors:** Lanjuan Xu, Tingting An, Chengjian Li, Xin Shi, Bo Yang

**Affiliations:** ^1^Department of Neurosurgery, The First Affiliated Hospital of Zhengzhou University, Zhengzhou, China 450052; ^2^Department of Critical Care Medicine, Zhengzhou Central Hospital Affiliated to Zhengzhou University, First Affiliated Hospital of Zhengzhou University, China 450001

## Abstract

**Objective:**

To assess the effect of adding coagulation indices to the currently existing prognostic prediction models of traumatic brain injury (TBI) in the prediction of outcome.

**Methods:**

A total of 210 TBI patients from 2017 to 2019 and 131 TBI patients in 2020 were selected for development and internal verification of the new model. The primary outcomes include death at 14 days and Glasgow Outcome Score (GOS) at 6 months. The performance of each model is evaluated by means of discrimination (area under the curve (AUC)), calibration (Hosmer-Lemeshow (H-L) goodness-of-fit test), and precision (Brier score).

**Results:**

The IMPACT Core model showed better prediction ability than the CRASH Basic model. Adding one coagulation index at a time to the IMPACT Core model, the new combined models IMPACT Core+FIB and IMPACT Core+APTT are optimal for the 6-month unfavorable outcome and 6-month mortality, respectively (AUC, 0.830 and 0.878). The new models were built based on the regression coefficients of the models. Internal verification indicated that for the prediction of 6-month unfavorable outcome and 6-month mortality, both the IMPACT Core+FIB model and the IMPACT Core+APTT model show better discrimination (AUC, 0.823 vs. 0.818 and 0.853 vs. 0.837), better calibration (HL, *p* = 0.114 and *p* = 0.317) and higher precision (Brier score, 0.148 vs. 0.141 and 0.147 vs. 0.164), respectively, than the original models.

**Conclusion:**

Our research shows that the combination of the traumatic brain injury prognostic models and coagulation indices can improve the 6-month outcome prediction of patients with TBI.

## 1. Introduction

The courses of traumatic brain injury (TBI) vary greatly among patients; thus, early prediction of outcomes is of great clinical value for clinical decision-making, personalized patient care, and research design [1]. The best two prognostic model established so far are international mission on prognosis and analysis of clinical trial (IMPACT) [2] and corticosteroid randomisation after significant head (CRASH) injury [3]. The two models predict the risk of 14-day mortality and 6-month outcome according to clinical, CT, and laboratory markers at admission. Because some hospitals in low-income countries may lack CT equipment, IMPACT Core, and CRASH Basic are the most valuable models. They have undergone external verification in high-income [4–6] middle-income, and low-income [7, 8] countries or regions since been established and need continuous development, improvement, and external verification to ensure universality in different environments.

Brain injury can cause coagulation disorder, which can predict the mortality risk of the patients [9, 10]. A meta-analysis showed [11] that coagulation disorder can increase the mortality of patients with TBI by 9 times and the probability of unfavorable prognosis by about 36 times. It is believed that coagulation disorder after head injury is an important independent risk factor affecting the prognosis of patients with TBI. Platelet count and the six items of coagulation are routine clinical testing indicators, which are easily available in Chinese hospitals. So far, there is no literature and report on the simultaneous verification of the two prediction models in the TBI population in Mainland China and the addition of any coagulation factors. Therefore, we had planned to verify the IMPACT Core model and the CRASH Basic model based on the existing data and screen out a more suitable prediction model. On this basis, new predictive factors such as coagulation indexes were added to develop new combined prognostic prediction models for TBI patients. Then, patients with TBI were recruited for verification of the new models. Finally, it is expected to provide value for clinical practice of patients with TBI in various situations and large-scale research design.

## 2. Methods

### 2.1. Patients and Data Collection

#### 2.1.1. Patients

Patients with TBI admitted to the Zhengzhou Central Hospital Affiliated to Zhengzhou University from 2017 to 2019 were retrospectively selected. Patients with TBI in 2020 were retrospectively collected. The clinical management of all patients is carried out in accordance with the latest version of the Guidelines for the Management of Severe Traumatic Brain Injury. [12]

Inclusion criteria were as follows: (1) at least 18 years old; (2) diagnosed as moderate to severe brain trauma by imaging (CT or MRI) and Glasgow Coma Score (GCS) is 3-12 points; and (3) within 12 hours of injury.

Exclusion criteria were as follows: (1) no blood samples were taken within 1 hour of admission; (2) patients with preexisting comorbidities that cause coagulation disorder or have been using anticoagulant drugs; (3) penetrating brain injury; and (4) pregnant women.

#### 2.1.2. Data Collection

According to the hospital's electronic medical records system, the patient's gender, age, cause of injury, time from injury to admission, hospitalization time, ICU time, GCS score at admission, vital signs, Abbreviated Injury Scale (AIS), and Injury Severity Score (ISS), whether there are severe extracranial injuries, pupil reactivity, emergency surgery situation, exercise score, hypoxia, hypotension, and first hemoglobin and coagulation indexes (APTT, INR, PT, platelets, and fibrinogen) on admission were analyzed.

The primary endpoint is death at 14 days, death at 6 months, and Glasgow Outcome Score (GOS) at 6 months. The GOS is mainly obtained by two staffs' telephone follow-up and discussion. It was divided into the following: (1) favorable prognosis: 5 points is good recovery and patient can live normally; 4 points is moderate disability, and patient can still live independently and work under protection; (2) unfavorable prognosis: 3 points is severe disability, patient unable to be independent in daily life; 2 points is vegetative state; 1 point is death.

### 2.2. The IMPACT and CRASH Models

#### 2.2.1. IMPACT

The IMPACT model adopted the data from 8 randomized controlled trials and 3 observational studies from 1984 to 1995 and was developed based on the impact factor of 8,509 patients aged ≥14 years with TBI and had a GCS score ≤ 12. It contains 3 parts: Core model: age, exercise score, and pupil reactivity to light; CT model: hypoxia, hypotension, CT rating, traumatic subarachnoid hemorrhage, and epidural hematoma were added based on the Core model; and lab model: laboratory indicators, namely, blood glucose and hemoglobin concentration, are added based on the CT model. Each model has two outcomes: 6-month mortality and 6-month unfavorable GOS outcome.

#### 2.2.2. CRASH

The CRASH prediction model was developed based on 1008 adult patients with TBI and GCS score ≤ 14 from 1999 to 2005. It contains 3 parts: Basic model: age, GCS, pupil reactivity, and whether there is a severe traumatic brain injury; CT model: the first CT scan results after injury were added. Each model has two outcomes: 14-day mortality and 6-month unfavorable GOS outcome.

### 2.3. Statistical Methods

Quantitative data are described by the mean ± standard deviation or median and interquartile range. Qualitative data are described by the number of cases and percentages. The 6-month mortality and the 6-month unfavorable outcome based on GOS were calculated according to the IMPACT Core model, while the 14-day mortality and the 6-month unfavorable outcome were calculated according to the CRASH Basic model. The area under the ROC curve (AUC) evaluates the predictive performance of the model, the Hosmer-Lemeshow (H-L) goodness-of-fit test evaluates the calibration of the model, and the Brier score measures the calibration in a quantitative way to obtain precision. In this paper, 6-month death is regarded as the outcome, single-factor logistic regression is used to screen variables related to the outcome, and the results are presented by OR and 95% CI. All statistical analyses were performed using SAS 9.4 software.

## 3. Results

### 3.1. Demographic and Clinical Features

359 patients with moderate to severe TBI met the inclusion criteria. Among them, 18 patients were lost during follow-up and could not be assessed for GOS. A total of 341 patients with moderate to severe TBI were included in the study, of which 210 from 2017 to 2019 were retrospectively screened into the development cohort, and 131 of 2020 were prospectively included into the validation cohort.  Table 1 shows the demographic features, relevant admission parameters, and 6-month outcome of the patients in the two groups. The 14-day mortality in the development cohort was 27.1%, which increased to 38.6% at 6 months. 59.5% of patients in the development cohort had unfavorable prognosis. The 14-day mortality rate in the validation cohort was 28.2%, which increased to 45.0% at 6 months. 73.3% of patients in the validation cohort had unfavorable prognosis.

### 3.2. The Development of Predictive Models

#### 3.2.1. The Performance of the IMPACT Core and CRASH Basic Models

The IMPACT Core model shows better discrimination and calibration than the CRASH Basic model.  Table 2 shows the 6-month unfavorable outcome (AUC = 0.807) and the 6-month mortality (AUC = 0.868) of the IMPACT Core model, and the calibration of both is good after H-L test (*p* > 0.05). Compared with the IMPACT Core model, the CRASH Basic model showed lower discrimination: 6-month unfavorable outcome (AUC = 0.766) and 14-day mortality (AUC = 0.791), and the calibration was not ideal (*p* < 0.05).

#### 3.2.2. Screening New Indicators to Develop New Models

With 6-month death as the outcome, the variables related to the outcome were screened through single-factor logistic regression. APTT, INR, PT, platelets, and fibrinogen were converted into binary variables based on reference value. In Table 3, PLT (OR = 0.99, *p* < 0.001), APTT (OR = 1.04, *p* = 0.006), INR (OR = 1.02, *p* = 0.024), and FIB (OR = 0.57, *p* = 0.001) show good correlations with the outcomes.

**Figure 1:**
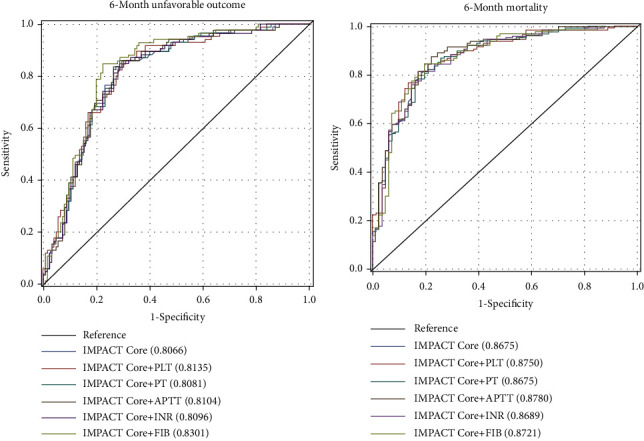
ROC curves of IMPACT Core and coagulation for 6-month adverse outcomes and mortality. (a) ROC curves for 6-month unfavorable outcome of IMPACT Core and coagulation. (b) ROC curves for 6-month mortality of IMPACT Core and coagulation.

Each time a coagulation index was added to the IMPACT Core model, the AUC and *R*^2^ of the logistic regression model for 6-month mortality and 6-month unfavorable outcome were calculated. In Figure 1 and Table 4, the highest AUC is the IMPACT Core+FIB model (AUC = 0.830) for the 6-month unfavorable outcome. The new model Logit_IMPACT Core+FIB_ = 0.9517 + 0.9797 × IMPACT Core − 0.5548 × FIB. For 6-month mortality, the highest AUC is the IMPACT Core+APTT model (AUC = 0.878). The new model Logit_IMPACT Core+APTT_ = −1.4747 + 1.4850 × IMPACT Core + 0.0348 × APTT.

### 3.3. The Performance Indicators of the New Combined Models

Table 5 shows the performance indicators of the new combined models. In terms of predicting 6-month unfavorable outcomes, the new combined model IMPACT Core+FIB shows a higher discrimination AUC (0.830 vs. 0.807). The H-L goodness-of-fit test shows that it has a good calibration in predicting 6-month unfavorable outcomes (*p* = 0.096). The Brier score shows a more satisfying precision (0.165 vs. 0.179). In terms of predicting 6-month mortality, the new combined model IMPACT Core+APTT shows a significantly higher discrimination ability AUC (0.878 vs. 0.868). The H-L goodness-of-fit test shows that it has a good calibration in predicting 6-month unfavorable outcome (*p* = 0.309). The Brier score shows a more satisfying precision (0.132 vs. 0.152).

### 3.4. Internal Verification of the New Combined Models

**Figure 2:**
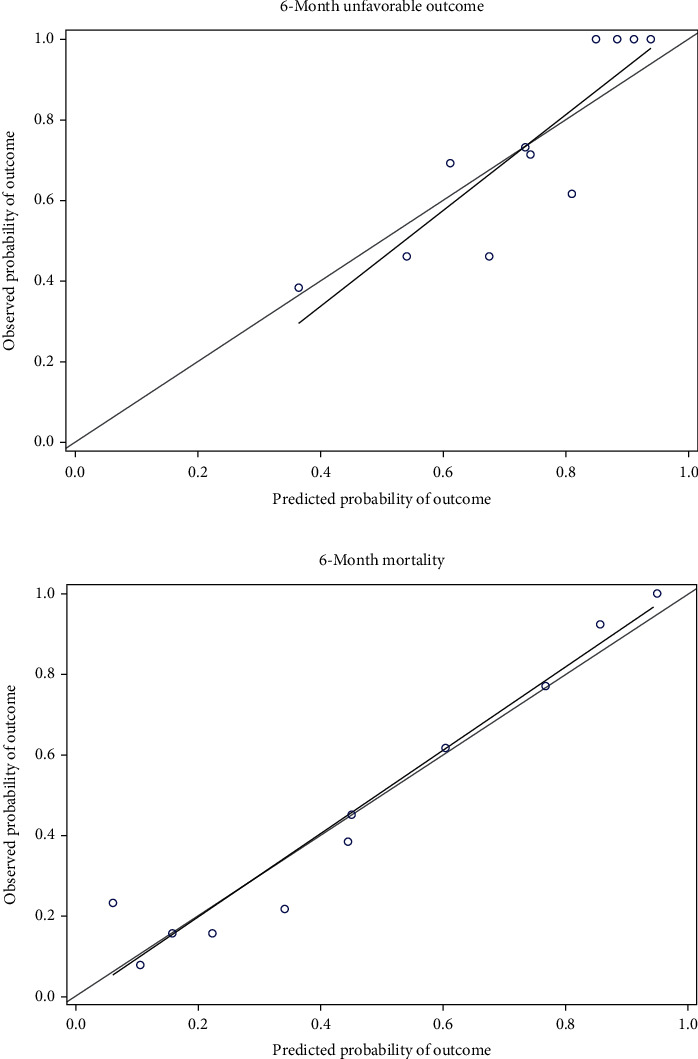
Calibration plots for 6-month adverse outcomes and mortality in the IMPACT core model. (a) Calibration plot of IMPACT Core+FIB to predict 6-month unfavorable outcome. (b) Calibration plot of IMPACT Core+APTT to predict 6-month mortality.

A total of 131 patients with TBI in 2020 were collected for internal verification of the new combined models.  Table 6 shows that its measurement indicators of internal verification performance are consistent with the results of Table 5. In terms of predicting 6-month unfavorable outcome, The IMPACT Core+FIB model shows higher discrimination AUC (0.823 vs. 0.818). The H-L goodness-of-fit test shows that it has a good calibration in predicting the 6-month unfavorable outcome (*p* = 0.114). The calibration chart (Figure 2(a)) shows that it is overestimated at lower scores and underestimated at higher scores. The Brier score shows a more satisfying precision (0.148 vs. 0.151).

In terms of predicting 6-month mortality, the new combined prediction model IMPACT Core+APTT shows better discrimination AUC (0.853 vs. 0.837). The H-L goodness-of-fit test shows that the new combined model has a good calibration in predicting 6-month mortality (*p* = 0.317). The calibration chart (Figure 2(b)) shows that the slope of the IMPACT Core+APTT model is close to 1, indicating that there is a strong consistency between the observed results and the predicted results. The Brier score shows a more satisfying precision (0.147 vs. 0.164).

## 4. Discussion

In this paper, we verified the predictive value of the IMPACT Core model and the CRASH Basic model in this population, screened out a better model, and developed a series of models combined with coagulation indicators. Our preliminary results show that adding coagulation indicators can improve the performance of the IMPACT Core model, and the results are still robust after internal reverification based on retrospective collection of data from such patient with TBI. It is learned that our study is the first study to simultaneously verify the IMPACT Core model and the CRASH Basic model in the TBI population in main land China, and the combination with coagulation factors has not been mentioned before. We proved that this combination's feasibility and sensitivity for 6-month prognostic prediction to patients with TBI, in order to help patients with TBI.

The IMPACT model and the CRASH model are developed by adopting the most advanced methods on large data sets. Our country has a vast territory, complex disease characteristics, and unbalanced medical resources. There is no literature or report about verification or improvement of these two prediction models in the TBI population of Mainland China. The predictive value of the IMPACT Core model and the CRASH Basic model in this population is demonstrated at the beginning of this paper and showed in Results of this paper. However, the IMPACT Core model shows better discrimination and calibration than the CRASH Basic model. Many studies have shown that [13] the CRASH model is preferred when the study target population includes patients from low-income and middle-income countries (LMICs) because the CRASH model has been developed on populations including a large number of LMIC patients, but the IMPACT model has been developed mainly on the population of a high-income country. The data in our study is collected from a first-class hospital at grade 3 in second-tier cities in China, and the performance of the IMPACT model outperforms the CRASH model. Recently, Wan et al. [14] used the IMPACT model to predict the 6-month unfavorable outcome (AUC = 0.80) and the 6-month mortality (AUC = 0.76) of the elderly with TBI aged ≥65 years in Mainland China. The value of the IMPACT model is proved in their study, which is consistent with our conclusions.

Previous studies have found that TBI-induced coagulation disorder (TBI-IC) is one of the important mechanisms for inducing secondary brain injury [15, 16]. Recently, Nakae et al. [17] showed the APTT is an independent predictor of unfavorable prognosis for patients with TBI. The multivariate analysis by Yuan et al. [18] corrected for factors such as age, GCS score, pupil response, and glucose and showed that APTT and INR are independent predictors of patients with TBI died in hospital. Overall, most studies have shown a mixture of early and delayed coagulopathy in TBI [19].The greatest risk factor for progression of hemorrhagic lesions was coagulopathy within the first 24 h after TBI [20]. Therefore, it is necessary to have a deeper understanding of the prognostic value of coagulopathy in the acute stage of TBI. Meanwhile, the results of the international mission on prognosis and analysis of clinical trials (IMPACT) in TBI study suggest that alterations in coagulation parameters, manifested by elevations in prothrombin time (PT) and decreased platelet count, may be reliable markers of TBI 6-month unfavorable outcome [21]. It is consistent with our conclusion that PLT, APTT, INR, and FIB are all strong predictors of 6-month death in patients with TBI. The above coagulation indicators are routine clinical testing indicators, which are easily available in Chinese hospitals. We added one coagulation index to the IMPACT core model variables each time and calculated that the IMPACT Core+FIB and IMPACT+APTT models scored the highest, respectively, for the 6-month unfavorable outcome and 6-month mortality. Ideally, a coagulation index will be selected ultimately, but our study screens two indexes FIB and APTT as predictors, respectively, of the new model for different outcomes, which is more accurate.

The new combined prediction models showed excellent discrimination for the 6-month unfavorable outcome and the 6-month mortality when they were internally revalidated for such TBI patients, which was higher than the previous report and showed good goodness-of-fit. The calibration chart in this paper shows that the IMPACT Core+FIB model is overestimated at low scores and underestimated at higher scores when predicting 6-month unfavorable outcome but generally has a good calibration, which is consistent with the Camarano et al.'s [22] calibration results of the IMPACT Core model recently. The slope of the IMPACT Core+APTT model is close to 1 when predicting 6-month mortality, indicating that there is a strong consistency between the observed results and the predicted results. The possible reason is that there are individual differences in many determinants such as socioeconomic status, family support, or acceptance of treatment when predicting the long-term functional prognosis of patients with TBI [23], but the new combined prediction models still show satisfying results in distinguishing patients with the highest probability of unfavorable outcomes based on only the data available at the time of admission. It is guessed that the final functional prognosis may be seriously affected by the early injury.

The advantage of this study lies in the simultaneous external verification of the IMPACT Core and CRASH Basic models in one part of China, and the predictive value showed for new populations. We combined the prognostic basic models of brain injury with coagulation indicators to further provide an idea and basis with evidence for the improvement of the models. The limitation of our research is that it is a single-center setting, so that the adequate calibration cannot be performed to distinguish effects of different center levels, and due to case mixed effects or continuous changes in patient epidemiology, we should evaluate the performance of newly developed models in different physical locations or clinical environments more frequently. External verification will be required in the future. We believe that our results will contribute to the development of tools that link clinical research and clinical data-based decision-making and encourage further research to enhance the predictive ability of current prognostic models in TBI. The ultimate goal is to improve the care of patients with head injuries.

## 5. Conclusions

Our research shows that IMPACT Core shows better predictive power than the CRASH Basic model. Each time a coagulation index was added to the IMPACT Core model, the new combined models IMPACT Core+FIB and IMPACT Core+APTT are the best for the 6-month unfavorable outcome and 6-month mortality, respectively. These results should be carefully evaluated in future prospective studies.

## Figures and Tables

**Table 1:** Basic information.

Variables	*N*	Test data set (2017-2019)	Validation data set (2020)
*N*	341	210	131
Gender (male)	341	146 (69.5)	97 (74.1)
Age (years), mean ± SD	341	54.0 ± 17.4	56.2 ± 15.4
Time from injury to admission (h), median (IQR)	341	1 (1-2)	1 (1-2)
GCS, median (IQR)	341	8 (4-11)	6 (3-10)
ISS, median (IQR)	341	26 (20-33)	26 (19-35)
AIS of head, median (IQR)	341	4 (3-4)	4 (4-5)
Severe traumatic brain injury, *n* (%)	341	92 (43.8)	59 (45.4)
Pupils, *n* (%)	341		
Both reactive		132 (62.9)	76 (58.0)
One reactive		10 (4.8)	2 (1.5)
Neither reactive		68 (32.4)	53 (40.5)
Motor score, *n* (%)	341		
Without reactivity		42 (20.0)	29 (22.1)
Hyperextension		29 (13.8)	13 (9.9)
Abnormal flexion		41 (19.5)	30 (22.9)
Normal flexion		54 (25.7)	28 (21.4)
Limitation		42 (20.0)	25 (19.1)
Obeys		2 (1.0)	6 (4.6)
Platelet (g/L), mean ± SD	341	176.5 (104-236)	185 (138-245)
Prothrombin time, PT (S), median (IQR)	341	11.5 (10.6-14.3)	11.5 (10.5-13.0)
Activated partial thromboplastin time, APTT (S), median (IQR)	341	23.9 (20.2-30.1)	27.1 (23.3-33.8)
International normalized ratio, INR, and median (IQR)	341	1.00 (0.92-1.25)	1.05 (0.96-1.15)
Fibrinogen (g/L), median (IQR)	341	1.96 (1.35-2.52)	1.89 (1.35-2.39)
14-day death, *n* (%)	341	57 (27.1)	37 (28.2)
6-month GOS outcome, *n* (%)	341		
Death		81 (38.6)	59 (45.0)
Vegetative state		13 (6.2)	18 (13.7)
Severe disability		31 (14.8)	19 (14.5)
Moderate disability		43 (20.5)	19 (14.5)
Favorable recovery		42 (20.0)	16 (12.2)
6-month death, *n* (%)	341	81 (38.6)	59 (45.0)
6-month outcome, *n* (%)	341	125 (59.5)	96 (73.3)

Abbreviations: GOS: Glasgow Outcome Scale; ISS: Injury Severity Score; AIS: Abbreviated Injury Scale.

**(a):** 

Models	6-month unfavorable outcome		6-month mortality	
	AUC (95% CI)	Hosmer-Lemeshow *χ*^2^ (*p* value)	AUC (95% CI)	Hosmer-Lemeshow *χ*^2^ (*p* value)
IMPACT Core	0.807 (0.747-0.866)	7.97 (0.437)	0.868 (0.816-0.919)	1.83 (0.986)

**(b):** 

Models	6-month unfavorable outcome		14-day mortality	
	AUC (95% CI)	Hosmer-Lemeshow *χ*^2^ (*p* value)	AUC (95% CI)	Hosmer-Lemeshow *χ*^2^ (*p* value)
CRASH Basic	0.747 (0.682-0.813)	20.19 (0.010)	0.791 (0.723-0.860)	20.47 (0.009)

Abbreviations: AUC: area under the receiver operating characteristic curve; CI: confidence interval.

**Table 3:** Univariate logistic regression.

Variable	OR	95% CI	*p*
Platelets (g/L)	0.99	0.99-1.00	<0.001
PT (S)	1.00	1.00-1.01	0.061
APTT (S)	1.04	1.01-1.06	0.006
INR	1.02	1.00-1.05	0.024
FIB (g/L)	0.57	0.41-0.80	0.001

Abbreviations: OR: odds ratio; CI: confidence interval.

**Table 4:** AUC of IMPACT and coagulation.

Models	6-month unfavorable outcome		6-month mortality	
	AUC (95% CI)	*R* ^2^	AUC (95% CI)	*R* ^2^
IMPACT Core	0.807 (0.747-0.866)	0.2579	0.868 (0.816-0.919)	0.3727
IMPACT Core+PLT	0.814 (0.755-0.872)	0.2746	0.875 (0.826-0.924)	0.3806
IMPACT Core+PT	0.808 (0.749-0.868)	0.2583	0.868 (0.816-0.919)	0.3736
IMPACT Core+APTT	0.810 (0.751-0.870)	0.2636	0.878 (0.829-0.927)	0.3895
IMPACT Core+INR	0.810 (0.751-0.869)	0.2628	0.869 (0.818-0.920)	0.3731
IMPACT Core+FIB	0.830 (0.774-0.886)	0.2931	0.872 (0.821-0.924)	0.3772

For the new model of 6-month unfavorable outcome: Logit_IMPACT Core+FIB_ = 0.9517 + 0.9797 × IMPACT Core − 0.5548 × FIB. For the new model of 6-month mortality: Logit_IMPACT Core+APTT_ = −1.4747 + 1.4850 × IMPACT Core + 0.0348 × APTT.

**Table 5:** Performance of models.

Variables	IMPACT Core	IMPACT Core+FIB	IMPACT Core	IMPACT Core+APTT
Outcome	6-month unfavorable outcome	6-month unfavorable outcome	6-month mortality	6-month mortality
AUC (95% CI)	0.807 (0.747-0.866)	0.830 (0.774-0.886)	0.868 (0.816-0.919)	0.878 (0.829-0.927)
Hosmer-Lemeshow (*p* value)	7.97 (0.437)	13.50 (0.096)	1.83 (0.986)	9.40 (0.309)
Brier score	0.179	0.165	0.152	0.132

**Table 6:** Validation of models.

Variables	IMPACT Core	IMPACT Core+FIB	IMPACT Core	IMPACT Core+APTT
Outcome	6-month unfavorable outcome	6-month unfavorable outcome	6-month mortality	6-month mortality
AUC (95% CI)	0.818 (0.746-0.891)	0.823 (0.754-0.892)	0.837 (0.763-0.911)	0.853 (0.782-0.923)
Hosmer-Lemeshow (*p* value)	8.17 (0.482)	12.94 (0.114)	1.52 (0.891)	9.30 (0.317)
Brier score	0.151	0.148	0.164	0.147

## Data Availability

The data used to support the findings of this study are included within the article.
